# Early-Life Development of the Intestinal Microbiome in Preterm and Term Infants Hospitalized in the Neonatal Intensive Care Unit

**DOI:** 10.3390/microorganisms13092158

**Published:** 2025-09-16

**Authors:** Jeongmin Shin, Chang Won Choi, Hyun Mi Kang, Sae Yun Kim, Young-Ah Youn

**Affiliations:** 1 Department of Pediatrics, Seoul St. Mary’s Hospital, College of Medicine, The Catholic University of Korea, 222 Banpo-daero, Seocho-gu, Seoul 06591, Republic of Korea; emmyshin@gmail.com (J.S.); pedhmk@catholic.ac.kr (H.M.K.); 2 Department of Pediatrics, Seoul National University College of Medicine, Seoul 03080, Republic of Korea; choicw1029@gmail.com; 3 Department of Pediatrics, Seoul National University Bundang Hospital, Seongnam 13620, Republic of Korea

**Keywords:** microbiome, very preterm, development

## Abstract

This prospective cohort study investigated the longitudinal compositional changes of the gut microbiome across different gestational age groups, from birth to six months’ corrected age for prematurity. Fecal samples (n = 709) from 349 neonates [51 very preterm, 195 moderate-to-late preterm, and 93 full-term infants] were analyzed. Proteobacteria, Firmicutes, and Bacteroidetes constituted the core microbiome of the meconium. Proteobacteria and Firmicutes were the dominant phyla before discharge, whereas Firmicutes was the most dominant phylum in all groups after discharge. *Ralstonia* was the most prevalent genus in the meconium of preterm infants. After discharge, the relative abundance of *Veillonella* continued to increase in all gestational groups (*p* = 0.011 for very preterm, *p* < 0.001 for moderate-to-late preterm and full-term). By six months corrected age, differences in the gut microbiota composition became less pronounced between the groups. The α-diversity of meconium was highest across all groups, and this significantly decreased during the neonatal intensive care unit stay and increased thereafter. The β-diversity was significantly different (*p* < 0.05) but of limited practical significance (*R*^2^ < 0.1). The differences between groups diminished as infants grew older, indicating that preterm infants were able to achieve a balanced gut microbiota and overcome dysbiosis.

## 1. Introduction

Preterm births are a major public health concern. Although advancements in medical technology have increased the survival rate of preterm infants, preterm neonates still face more challenges than those faced by term infants. This is mainly because of their immaturity, which may pose an increased risk of morbidity and mortality and lead to diseases, such as necrotizing enterocolitis (NEC) [1] and sepsis [2,3]. Preterm morbidities are closely associated with intestinal microbiota. Stewart et al. reported that pathogens isolated in cases of late-onset sepsis are commonly found in the gut microbiota [4]. Kang et al. revealed that infants diagnosed with NEC or feeding intolerance exhibited a substantially lower abundance of Bacteroidetes and a higher abundance of Firmicutes and other microbes than those exhibited by infants discharged from the neonatal intensive care unit (NICU) without any gastrointestinal symptoms [5]. Furthermore, Beghetti et al. suggested that early *Bifidobacterium* deficiency is associated with adverse neurological outcomes in very low birth weight (VLBW) infants [6], and early dysbiosis may interfere with microbiota metabolic capacity. Consequently, this alters nutrient absorption and influences growth and neurodevelopment [7,8].

Traditionally, the fetal environment is believed to be entirely sterile [9]; however, the “in utero colonization” hypothesis challenges this opinion. This hypothesis suggests that the in utero environment is not sterile, and the first passed meconium may reflect the in utero microbial habitat [10,11]. Therefore, postnatal events influence microbial exposure in infants, leading to the establishment of distinct gut microbiota compositions [12,13]. The intestinal microbiota plays a crucial role in the maturation of the host immune system by regulating immune responses, protecting against opportunistic pathogen invasion, and modulating intestinal endocrine function, all of which have crucial health implications [14,15,16].

Owing to their immaturity, preterm infants tend to stay in the NICU for prolonged durations [17]. Extended hospitalization affects the development and stability of the gut microbiota, which interrupts the establishment of a mature gut microbiota in infants [18]. Gestational age (GA) is the primary determinant of gut microbiota development in neonates [19,20]. Compared with that of full-term (FT) infants, preterm infants exhibit altered gut microbiota. Furthermore, dysbiosis was discovered in the first-passed meconium of preterm infants compared with that of FT infants [5,21,22], which is in line with previous studies [23,24].

Growing evidence links the gut microbiome to health risks in the pediatric population [25]. However, few studies have conducted longitudinal analyses of the preterm gut microbiome, including follow-up after hospitalization. In this study, meconium and fecal samples were collected from preterm infants with varying GAs spanning from the neonatal period to six months corrected age for prematurity (CA). This study aimed to assess the dynamic changes in the gut microbiota of fecal samples collected from infants with different GAs. Specifically, this study aimed to describe the differences in the microbiota composition and diversity between very preterm (VP), moderate-to-late preterm (MLP), and FT neonates at each time point. Additionally, this study sought to observe longitudinal changes in the microbiota composition within each GA group over time in a large cohort of Korean neonates.

## 2. Materials and Methods

### 2.1. Study Population

This prospective cohort study was conducted at the Catholic University of Korea, Seoul St. Mary’s Hospital, a tertiary referral university hospital with a level IV NICU, located in Seoul, Republic of Korea. All participants were recruited from a single urban center. Written informed consent was obtained from the parents of all the enrolled newborns. This study was conducted in accordance with the Declaration of Helsinki, and the protocol was approved by the Human Research Ethics Board at Seoul St. Mary’s Hospital (KC22RISI0240, approved on 14 April 2022; KC22TNSI0297, approved on 3 June 2022) and performed in accordance with the relevant guidelines and regulations.

**Figure 1:**
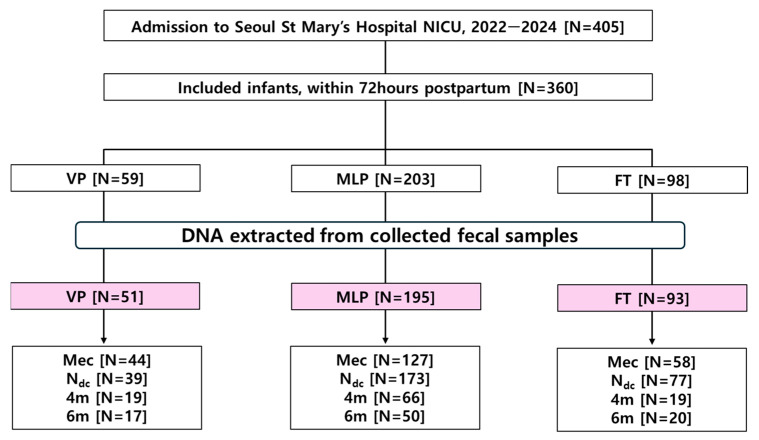
Flow chart of the study cohort. Participants were classified into three groups according to their gestational age at birth: VP (<32 weeks), MLP (32–37 weeks), and FT (>37 weeks). Meconium samples (Mec) and the last stool before NICU discharge were collected as the second sample (N_dc_). The third (4m) and fourth (6m) samples were collected after NICU discharge: at 4 months of age and 6 months of age (or corrected age for prematurity). Abbreviations: 4m, stool samples collected at 4 months of corrected age for prematurity; 6m, stool samples collected at 6 months of corrected age for prematurity; FT, full-term infant; Mec, meconium; MLP, moderate to late preterm; N_dc_, last stool before NICU discharge; NICU, neonatal intensive care unit; VP, very preterm.

All neonates admitted to the NICU between April 2022 and January 2024 were eligible for inclusion. The inclusion criteria were as follows: (1) admission to the NICU regardless of GA at birth and (2) provision of written informed consent by legal guardians. Infants with major congenital abnormalities, syndromes, or metabolic diseases were excluded. In the FT group, infants primarily admitted because of gastrointestinal problems were excluded. Participants were categorized into three groups based on their GA at birth: VP (<32 weeks), MLP (32–37 weeks), and FT (37–42 weeks). A flowchart of this study is shown in Figure 1.

The clinical course and disease progression of the infants were surveyed using an electronic medical record system. Baseline information, including maternal factors (age, obstetric complications, and mode of delivery), GA, birth weight, perinatal history, major hospital outcomes, and post-discharge clinical conditions, was collected from both mothers and infants.

### 2.2. Sample Collection and Analysis Method

#### 2.2.1. Sample Collection

Fecal stool samples were collected at four different time points. Meconium [Mec] reflects the intrauterine environment, whereas the last stool sample collected before NICU discharge [N_dc_] indicates the influence of the NICU environment on the infant gut microbiome. Fecal samples collected at four months CA [4m] are influenced by the home or community environment, whereas those collected at six months CA [6m] reflect the changes that occur following the introduction of a weaning diet.

First, the Mec samples were collected within 72 h of birth. Second, N_dc_ fecal samples were collected 1–3 days before discharge from the NICU. After discharge, the third and fourth fecal samples were collected at home, using a stool collection kit with special transport medium (REST™ NBgene-GUT, Noblebiosciences, Hwaseong, Republic of Korea): four months CA (four months for FT group, 4m) and six months CA (six months for FT group, 6m). The NICU nurses collected the Mec and N_dc_ samples as part of their routine care. Immediately after passage, 1–2 mL of infant stool was collected from diapers using sterile spatulas, placed into sterile tubes by nurses, and stored at −20 °C. The 4m and 6m samples were collected at home by the parents, stored at room temperature (15–25 °C), and delivered to the laboratory (AIBIOTICS Co., Ltd., Changwon, Republic of Korea) within 14 days of collection. Upon arrival, the specimens were stored at −80 °C until DNA was extracted for microbiome analysis.

#### 2.2.2. Fecal Microbiome Analysis Method: 16S rRNA Sequencing and Processing

DNA was extracted from fecal samples, and PCR amplification was performed by targeting the V3–V4 region of the 16S rRNA gene. Subsequently, an index PCR was conducted to attach dual indices using PCRBIO VeriFi Mix (PCR Biosystems^®^, London, UK) and the Nextera^®^ Index Kit V2 Set A (Illumina^®^, San Diego, CA, USA). After indexing, the final pooled library was assessed for its concentration and fragment size. DNA libraries were pooled and sequenced on an Illumina MiSeq platform. Sequencing data of the V3–V4 variable regions were analyzed using the 16S Metagenomics App. Detailed sampling, preprocessing, and analysis methods for the meconium have been published elsewhere [5,21].

### 2.3. Definitions

GA at birth was assessed based on the last menstrual period or ultrasound findings during the first trimester. Antenatal corticosteroids (ACSs) were defined as the administration of more than one dose of ACS to the mother before delivery for fetal lung maturity. Premature rupture of membranes (PROMs) was defined as rupture that lasted for >18 h before delivery. Histological chorioamnionitis was defined according to Yoon et al. [26] as the presence of acute inflammatory changes in any placenta-related tissue sample. Small-for-gestational-age was defined as a birth weight below the 10th percentile for GA and sex [27]. Respiratory distress syndrome (RDS) was diagnosed based on both clinical and radiographic findings. Bronchopulmonary dysplasia (BPD) was classified according to the National Institute of Child Health consensus on BPD severity [28]. Sepsis was determined based on positive blood culture results. NEC stage II or higher was also reviewed [29]. Feeding intolerance was defined as persistent gastric aspirates exceeding 50% of the feed volume, with or without increased abdominal girth, in the absence of sepsis or radiographic evidence of NEC, lasting for 48 h and occurring more than three times per day, thereby preventing the advancement of feeding beyond 10–20 mL/kg/day [30]. Retinopathy of prematurity was defined as stage 3 or higher and/or requiring treatment [31,32].

CA (corrected age for prematurity) was used for preterm infants, calculated by subtracting the number of weeks born before 40 weeks of gestation from the chronological age. This adjustment is based on the expected full-term due date and refers to the actual time elapsed since birth.

### 2.4. Statistical Analyses

Clinical characteristics and microbiological data were compared among VP, MLP, and FT infants. Continuous variables were summarized as means with standard deviations or as medians with interquartile ranges (IQRs), whereas categorical variables were presented as frequencies and percentages, as appropriate. One-way analysis of variance was conducted using the post-hoc Bonferroni correction method. Alternatively, the Kruskal–Wallis test and additional pairwise comparison for post-hoc analyses were performed if the normality of the variable was not confirmed. Differences in categorical variables were compared using the chi-square test for trends or Fisher’s exact test. Alpha diversity was calculated using Shannon’s diversity index, and beta diversity was plotted using principal coordinate analysis (PCoA) of Bray–Curtis dissimilarity. All statistical tests were two-sided, and differences between group correlations were considered significant at *p* < 0.05. All statistical analyses were performed using SPSS version 24 software (IBM Corp., Armonk, NY, USA) and the R software package version 4.3.0 (R Foundation for Statistical Computing, Vienna, Austria).

## 3. Results

### 3.1. Study Cohort, Demographic, and Clinical Information

A total of 360 neonates fulfilled all inclusion criteria, and stool samples were collected. After preprocessing, DNA was extracted for microbiome analyses from 709 samples from 339 infants: 51 VP infants, 195 MLP infants, and 93 FT infants (Figure 1).

The basic characteristics of the infants and their mothers were analyzed (Table 1). The mean GA at birth for the FT group was 37.9 ± 1.0 weeks, and the mean birth weight was 3160.2 ± 412.5 g. Among the preterm infants, the mean GA and birth weight of the MLP group were 34.1 ± 1.2 weeks and 2186.7 ± 429.7 g, respectively. In contrast, the mean GA and birth weight of the VP group were 28.0 ± 2.6 weeks and 1125 ± 453.5 g, respectively.

The method of conception (natural versus assisted reproduction) differed significantly between the three groups (*p* < 0.001). The proportion of ACS administrations decreased with increasing GA in the VP (96.1%), MLP (77.9%), and FT (1.1%) groups (*p* for trend < 0.001). The proportion of pregnancy-related maternal complications, such as PROM, histological chorioamnionitis, maternal diabetes mellitus, and maternal preeclampsia, also decreased significantly with increasing GA (*p* for trend < 0.05). The proportion of infants delivered via Cesarean section was significantly higher in the VP (96.1%) and MLP (96.9%) preterm groups than in the FT group (78.5%). Preterm infants more frequently required intubation at birth: 52.9% for VP, 3.9% for MLP, and 2.2% for FT (*p* for trend < 0.001). Younger GA infants tended to develop RDS more easily (82.4% for the VP, 25.6% for the MLP, and 17.2% for the FT groups; *p* for trend < 0.001). The proportion of infants who developed NEC or feeding intolerance decreased with maturity: 23.5%, 10.3%, and 3.2% in the VP, MLP, and FT groups, respectively (*p* for trend = 0.001). Sepsis showed a similar decreasing pattern based on GA, with rates of 17.6%, 0.5%, and 0.0% in the VP, MLP, and FT groups, respectively (*p* for trend < 0.001).

### 3.2. Structure of the Gut Microbiota

Fecal samples were collected from as many infants as possible at each time point, resulting in a total of 812 samples. After excluding those with poor preservation or inadequate sequencing quality, 709 samples were used for DNA extraction and sequencing analyses. The gut microbiota composition of preterm infants with different GAs was compared at various days after birth.

#### 3.2.1. Meconium Stage (Mec Samples)

**Figure 2:**
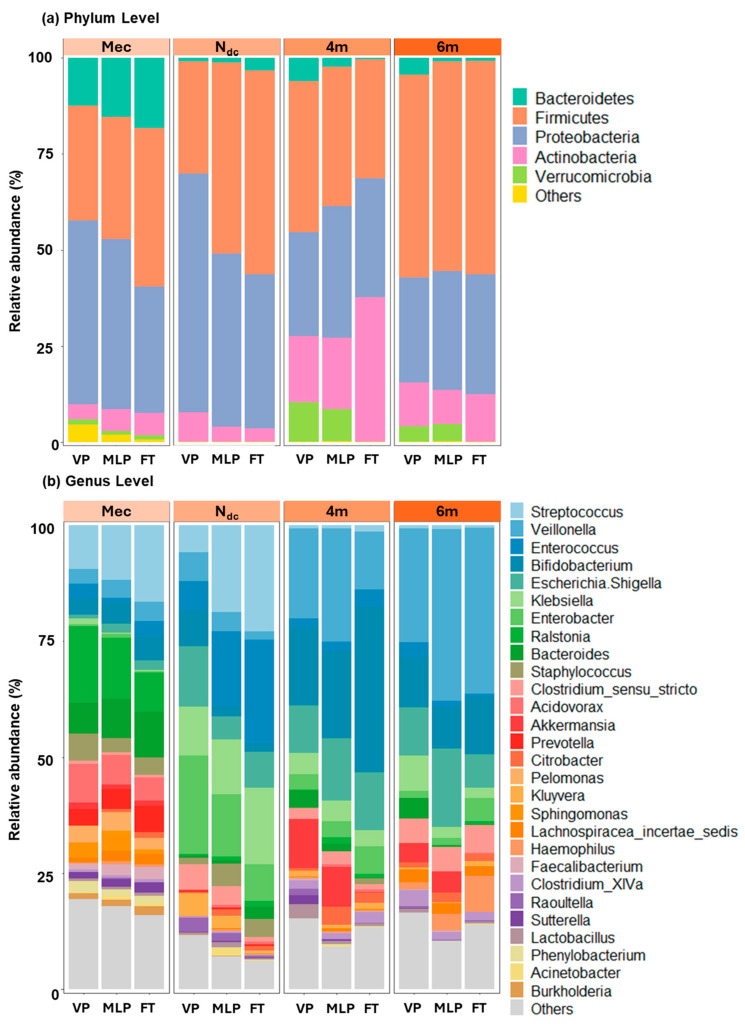
Development of infantile gut microbiota composition from birth to 6 months of corrected age for prematurity according to gestational age. This figure shows the relative abundance at the (**a**) phylum level, (**b**) genus level at birth (Mec), last stool before NICU discharge (N_dc_), 4 months of corrected age for prematurity (4m), and 6 months of corrected age for prematurity (6m). The different colors correspond to different species names, and the length of the color block indicates the relative abundance of the species represented by the color block. The abscissa represents the different sampling time points, and the comparison between very preterm, moderate-to-late preterm, and full-term neonates is described separately at each time point. The ordinate is the relative abundance of the species. Abbreviations: 4m, stool samples collected at 4 months of corrected age for prematurity; 6m, stool samples collected at 6 months of corrected age for prematurity; FT, full-term infants; Mec, meconium; MLP, moderate to late preterm; N_dc_, last stool before NICU discharge; NICU, neonatal intensive care unit; VP, very preterm.

**Figure A1:**
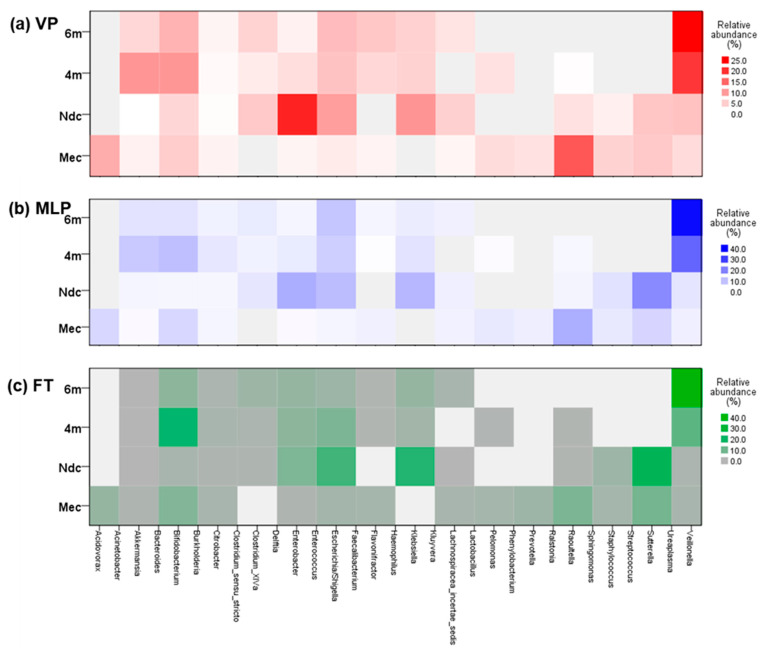
Heat map of the core microbiome at the genus level from the infant fecal samples collected at four different time points. Meconium (Mec), the last stool sample before NICU discharge (N_dc_), and stool samples collected at 4 months (4m) or 6 months (6m) of corrected age (for preterm infants) or chronological age (for term infants). Abbreviations: FT, full-term infants; MLP, moderate to late preterm; N_dc_, last stool before NICU discharge; NICU, neonatal intensive care unit; VP, very preterm.

The three most abundant bacterial phyla in each group were Proteobacteria, Firmicutes, and Bacteroidetes. The relative abundance of Proteobacteria was 44.2–47.8% in preterm infants and 32.9% in meconium for FT infants. The relative abundances of Proteobacteria and Firmicutes differed significantly among the three groups (*p* = 0.007 and *p* = 0.032, respectively). At the genus level, the composition varied greatly among the groups. The three most abundant genera in the VP group were *Ralstonia* (16.5%), *Streptococcus* (9.4%), and *Acidovorax* (8.3%). In the MLP group, these were *Ralstonia* (13.2%), *Streptococcus* (11.7%), and *Bacteroides* (8.5%), whereas, in the FT group, these were *Streptococcus* (16.5%), *Bacteroides* (9.8%), and *Ralstonia* (8.5%) (Figure 2 and Figure A1, Tables S1 and S2).

#### 3.2.2. Changes During NICU Admission (N_dc_ Samples)

The fecal microbiota of the preterm and FT groups at the end of NICU admission was analyzed. The composition of the gut microbiota in the N_dc_ group was altered (Figure 2).

The three most abundant phyla were the Proteobacteria, Firmicutes, and Actinobacteria. Proteobacteria was the most abundant phylum in the VP group (62.3% in VP, 45.1% in MLP, and 40.1% in FT groups, *p* = 0.010). However, Firmicutes was the most abundant phylum in the N_dc_ samples of the MLP and FT groups. The relative abundance of Firmicutes increased with increasing GA (29.0% in VP, 49.6% in MLP, and 53.0% in FT, *p* = 0.002). The third most abundant phylum was Actinobacteria (7.6% in VP, 3.8% in MLP, and 3.4% in FT; *p* = 0.074). At the genus level, the three most abundant genera in the VP group were *Enterobacter* (21.2%), *Escherichia/Shigella* (13.0%, and *Klebsiella* (10.6%). The most abundant genera in the MLP group were *Streptococcus* (18.6%), *Enterococcus* (16.3%), and *Enterobacter* (13.4%). In the FT group, these bacteria included *Streptococcus* (22.9%), *Enterococcus* (22.4%), and *Klebsiella* (16.6%). The relative abundances of *Streptococcus*, *Enterococcus*, and *Enterobacter* were significantly different between the three groups (*p* = 0.002, 0.006, and 0.018, respectively). *Bifidobacterium* was the next most common taxon in the VP group (7.6%); however, its abundance was lower in the MLP and FT groups (2.1% and 1.7%, respectively; *p* = 0.001). Significant differences in the abundance of *Kluyvera*, *Raoultella*, and *Bacteroides* were observed among the three groups (Figure 2 and Figure A1, and Table S2).

#### 3.2.3. Changes After NICU Discharge (4m and 6m Samples)

After discharge, the relative abundance of *Veillonella* continued to increase in all gestational groups (*p* = 0.011 for very preterm, *p* < 0.001 for moderate-to-late preterm and full-term). By six months corrected age, differences in the gut microbiota composition became less pronounced between the groups (Table S3).

### 3.3. Microbial Diversities

**Figure 3:**
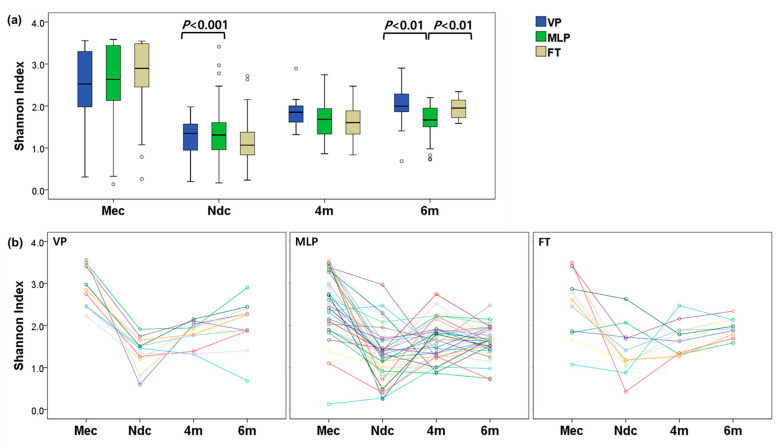
*α*-diversities in infantile gut microbiome. The gut microbiome was analyzed at four time points: meconium (Mec), the last stool sample before NICU discharge (N_dc_), and stool samples collected at 4 months (4m) or 6 months (6m) of corrected age (for preterm infants) or chronological age (for term infants). Alpha diversity was assessed using the Shannon diversity index. (**a**) Box-and-whisker plots show the distribution of Shannon indices across different gestational age groups at each time point. Median values were compared using the Kruskal–Wallis test (non-parametric analysis of variance). Boxes represent interquartile ranges, horizontal lines indicate medians, and whiskers represent the full range of values. (**b**) Spaghetti plots depict the longitudinal changes in Shannon diversity for individual infants. Each colored line represents one subject’s trajectory across available time points within each gestational age group. Abbreviations: FT, full-term infants; MLP, moderate to late preterm; N_dc_, last stool before NICU discharge; NICU, neonatal intensive care unit; VP, very preterm.

Compared with that of the FT group, the meconium of preterm infants showed lower α-diversity. The median (IQR) Shannon index values were 2.523 (1.976–3.339), 2.633 (2.104–3.444), and 2.895 (2.445–3.487) for the VP, MLP, and FT groups, respectively; however, these differences were not statistically significant. The α-diversities decreased during NICU admission. The Shannon indices of the N_dc_ samples were 1.345 (0.938–1.601) for the VP group, 1.308 (0.954–1.618) for the MLP group, and 1.064 (0.823–1.391) for the FT group, respectively, with a significant difference between the MLP and FT groups (*p* = 0.010). The Shannon indices slightly increased after NICU discharge, with values of 1.849 (1.574–2.028) in the 4m samples and 1.995 (1.844–2.344) in the 6m samples for the VP group. The differences among the three groups for 4m samples were not statistically significant. However, in the 6m samples, the differences in Shannon indices between the VP and MLP groups were significant (*p* = 0.001). In terms of temporal changes in α-diversities, the three groups showed similar patterns. The Shannon index in stools was highest in the Mec samples, and the α-diversity measure markedly decreased in the N_dc_ samples. After discharge from the NICU, the Shannon index slowly increased (Figure 3; Tables S4 and S5).

Quantification of the compositional dissimilarity (β-diversity) via the Bray–Curtis dissimilarity index showed a shift according to GA from the VP to the FT group. A Bray–Curtis dissimilarity matrix was calculated for each sample to compare the bacterial composition of each GA group.

**Figure 4:**
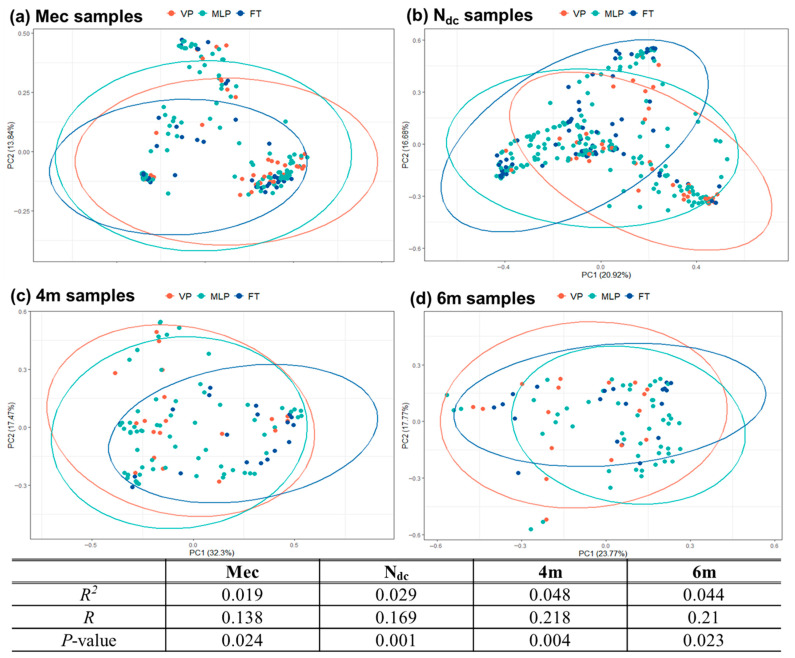
*β*-diversities of infantile gut microbiota. *β*-diversities were evaluated by Bray–Curtis dissimilarity. Principal coordinate analysis (PCoA) reflects the distribution of the different samples: meconium (*R*^2^ = 0.019, *p* = 0.024) (**a**), last stool before NICU discharge (*R*^2^ = 0.029, R = 0.017, *p* = 0.001) (**b**), 4 months of corrected age (*R*^2^ = 0.048, *p* = 0.004) (**c**), and 6 months of corrected age (*R*^2^ = 0.044, *p* = 0.023) (**d**). Each point represents one sample and is colored based on the group. Abbreviations: 4m, stool samples collected at 4 months of corrected age for prematurity; 6m, stool samples collected at 6 months of corrected age for prematurity; FT, full-term infants; Mec, meconium; MLP, moderate to late preterm; N_dc_, last stool before NICU discharge; NICU, neonatal intensive care unit; VP, very preterm.

PCoA plots (Figure 4) revealed the distances between the bacterial communities in all individual samples. For the Mec samples, the contribution rates were 43.99% and 13.84% for the first and second principal components, respectively, whereas those for the N_dc_ samples were 20.92% and 16.68% for the first and second principal components, respectively. The Bray–Curtis dissimilarity scores between samples indicated that the community structure of the meconium could not be clearly differentiated according to the GA (*R* = 0.138 and *p* = 0.024 for Mec; *R* = 0.169 and *p* = 0.001 for N_dc_). The Bray–Curtis distance between samples showed that the community structure at 4m and 6m could be more clearly distinguished (*R* = 0.218, *p* = 0.004 for 4m; *R* = 0.210, *p* = 0.023).

## 4. Discussion

The gut microbiota plays crucial roles in various aspects of health and disease. This study contributes substantially to the current understanding of the developmental changes in the gut microbiota of Korean preterm and FT infants with different GAs. The core microbiome consisted of Proteobacteria, Firmicutes, Bacteroidetes, and Actinobacteria, which were the most abundant phyla until six months of CA. However, temporal changes in the composition of the microbiome were observed in each group. Second, the gut microbiota varies according to the GA, with the most noticeable differences occurring during NICU admission. Finally, the differences between the groups, particularly in the VP group, diminished as the infants grew, thus reflecting a shift from dysbiosis in the microbiome of VP infants toward a state of symbiosis.

### 4.1. Dynamic Changes in NICU Were Exaggerated in VP Group

The hospital microbiome encompasses persistent microbial communities residing on surfaces, medical equipment, healthcare providers, and in the air, all of which can contribute to early gut colonization in hospitalized infants [33]. Preterm infants in the NICU are frequently exposed to multiple prenatal and postnatal inflammatory influences—such as maternal chorioamnionitis, formula feeding, invasive procedures, and the administration of antibiotics or other medications [34], collectively referred to as NICU microbiome exposure. Exposure to these insults may influence the gut microbiome development in preterm infants. Compared with that in FT infants, preterm neonates exhibit decreased initial gut microbiome diversity and disruption of core microbiome composition [21,35,36]. Furthermore, they develop a distinct gut microbiota characterized by delayed colonization and reduced diversity during NICU admission compared with that in FT infants [37].

Dysbiosis of microbial succession in VP/VLBW infants is likely to increase the risk of infection and inflammatory processes. Additionally, the immature immune system of preterm infants contributes to reduced resistance to pathogens, which may lead to intestinal complications, such as feeding intolerance, increased incidence of NEC, and other related morbidities [5,38]. Proteobacteria are frequently found in high abundance during disease and have been proposed as markers of microbial instability, potentially predisposing the host to disease onset [39]. Their increased abundance is associated with host inflammatory responses and has been extensively studied in mouse models of colitis [40]. Both late-onset sepsis and NEC were associated with microbiomes dominated by Proteobacteria [41]. According to a meta-analysis by Pammi et al., infants with NEC consistently showed an increased relative abundance of Proteobacteria and a decreased abundance of Firmicutes and Bacteroidetes [42]. Similarly, neonates with sepsis in India exhibited pronounced gut microbiome dysbiosis characterized by elevated Proteobacteria levels [43]. Furthermore, the abundance of Proteobacteria varies with GA and postnatal age. Jia et al. found that Proteobacteria levels were elevated in the meconium of extremely preterm or VP infants and decreased over the course of NICU hospitalization [44]. The study findings are consistent with this pattern; the relative abundance of Proteobacteria increased markedly during NICU admission (N_dc_) and decreased after discharge, whereas the proportions in the MLP and FT groups remained relatively stable throughout the sampling period.

At the genus level, the meconium of FT infants was predominantly colonized by bacteria from the digestive tract, whereas preterm infants were more frequently colonized by bacteria from the skin and hospital environment. The study cohort exhibited similar differences. In the microbiome of FT infants, *Streptococcus* and *Bacteroides* were the two most abundant genera. In contrast, *Ralstonia* was the most common genus identified in the meconium microbiome of the preterm infants. *Ralstonia* spp. are environmental microorganisms that are known for their remarkable ability to persist in medical equipment and solutions [45]. Abundant *Ralstonia* has often been observed in mothers with gestational morbidities, such as chorioamnionitis [46], which poses a risk of preterm delivery. These mothers frequently experience prolonged hospitalization and are treated with antibiotics that may alter their microbiota as well as that of the fetus. These factors may have contributed to the differences in meconium microbiome composition between term and preterm infants in this study cohort. Although *Ralstonia* is a potential contaminant in DNA extraction kits and sequencing reagents, its consistent and differential abundance across the GA groups in this study suggests a true biological signal rather than reagent-derived contamination.

This study revealed that α-diversity was highest in meconium samples and declined thereafter, thereby challenging the notion of a sterile intrauterine environment. Maternal microbial transmission occurs before birth via the placenta, amniotic fluid, or immune cells, thus supporting the concept of intrauterine seeding [10,47]. Despite the high cesarean section rate in our cohort, the Mec samples showed unexpectedly rich microbial diversity. This suggests that initial colonization may not depend solely on vaginal exposure during delivery but may also depend on prenatal maternal factors. Although cesarean delivery reduces the transmission of vaginal microbes [48,49], the study findings underscore the role of intrauterine and maternal influences in shaping the neonatal microbiome. Further research is needed to identify modifiable maternal factors and explore prenatal interventions to promote healthy microbial colonization, especially in infants at risk of prematurity or dysbiosis.

### 4.2. Development of Gut Microbiome in VP Infants During Early Home Period

The early home period after birth is an obstetric term typically lasting from 0 to 3 months. For preterm infants admitted to the NICU, the early home period began after hospital discharge. The hospital environment is likely to differ considerably from the home in terms of resident microbes and microbial resistance.

During this period, the gut microbiome of preterm infants continues to develop and undergo pronounced changes to adapt to the new environment and diet. Eventually, the gut microbiome becomes more similar to that of FT infants [50]. Common findings observed across the three groups included an increase in the relative abundance of Actinobacteria in the N_dc_ and 4m samples. Actinobacteria are core microorganisms in the gut microbiota and are also found in the skin, oral cavity, and genitourinary tract [51]. They form diverse symbiotic interactions that are often beneficial to gut health and contribute to gut homeostasis, immune system function, and the digestion of complex carbohydrates [51,52,53]. Antibiotic exposure potentially reduces the abundance of Actinobacteria, particularly in early life [54]. In healthy infants who adjusted to a home environment without antibiotics, the abundance of Actinobacteria increased after the early home period. Actinobacteria was the most abundant phyla in the FT group, whereas in the preterm group, it was the third most common taxon in the 4m stool samples. These differences disappeared in the 6m stool samples, indicating that the microbiome of preterm infants matured with age and became more similar to that of FT infants.

### 4.3. Adaptation After Initiating the Weaning Diet

The gut microbiota of the VP group gradually converged with that of FT infants six months of CA. The top three phyla and top two genera were the same between the VP and FT groups, indicating a similarity in bacterial composition, although the order of abundance differed. As infants grow, they begin to consume solid food and transition to weaning diets. During the weaning process, the introduction of various solid foods and novel dietary components leads to an increase in microbial α-diversity within the gut microbiome [55]. In South Korea, infants typically begin to wean off breast milk or formula at 4 and 6 months of age. The changes observed in the microbiomes between the 4m and 6m stool samples reflected a shift in the dominant taxa as a result of the weaning diet.

*Akkermansia*, a genus belonging to the phylum Verrucomicrobia, showed an increase in relative abundance in the preterm groups in the 4m and 6m stool samples compared to the N_dc_ samples. Verrucomicrobia plays a critical role in maintaining gut barrier integrity and modulating immune responses and has been associated with various health benefits in adults, including improved metabolic function and anti-inflammatory effects [56]. Consistent with our results, Verrucomicrobia was frequently detected in the gut microbiota of preterm infants, whereas it was less prevalent in FT infants. This difference is particularly notable in infants who receive total parenteral nutrition with delayed initiation of enteral feeding, as these conditions create a unique gut environment that favors colonization by Verrucomicrobia [57]. In VP infants, prolonged reliance on parenteral nutrition is likely to contribute to the distinct microbial composition observed in this group.

Changes were observed at the genus level after initiation of the weaning diet. Similar to previous studies [55,58], *Veillonella*, *Escherichia/Shigella*, and *Bifidobacterium* were the top three genera in the microbiome in 6m stool samples. Notably, *Veillonella* continued to increase in both the preterm and FT groups, with the most distinctive increase in relative abundance between the 4m and 6m stool samples. *Veillonella* belongs to the phylum Firmicutes, and its primary function is to ferment lactate into short-chain fatty acids, which regulate the pH within the colon, promote a healthy environment for other gut bacteria, and serve as an energy source for the host [59]. *Veillonella* increases in abundance following the introduction of solid foods during weaning, signifying the natural maturation of the infant gut microbiome [60]. This shift aligns with our results and is associated with the transition from the meconium to normal stool, thereby reflecting a more mature gut microbiome.

### 4.4. Decreased Diversity

In adults and children, lower α-diversity is a marker of dysbiosis, whereas increased α-diversity is associated with the stability of the microbial community and colonization. Compared with that of FT infants, the gut microbiota of preterm infants is characterized by decreased microbial diversity [8,21,61]. In this study, the Shannon index for α-diversity in the meconium increased as GA advanced, although the change was not statistically significant. VP infants develop a markedly different and sparse microbiome compared with that of FT infants.

In an additional analysis examining the temporal changes within each group, the trends were similar across the three groups. The Shannon indices were highest in the Mec samples, followed by a sharp decrease in the last stool samples collected in the NICU (Ndc). However, given the low-biomass nature of meconium [62], technical contamination during sample collection, DNA extraction, or sequencing must always be considered. Furthermore, α-diversity should be carefully interpreted. After discharge, the α-diversity of the gut microbiome appeared to gradually increase. Increased α-diversity indicates a greater variety of organisms present, with each bacterium being more evenly distributed.

Although the microbiome composition at each time point differed depending on GA at birth, the rate of development varied. However, the direction of development remained consistent. Although gut dysbiosis in preterm infants temporarily affects the variety and diversity of the microbiome [21], the intestinal microbiome of preterm infants may begin its trajectory toward maturation after NICU discharge.

Beta-diversity analyses revealed significant differences in the microbial community composition across all four time points (*p* < 0.05); however, low *R*^2^ values indicated small effect sizes. This suggests that although GA was associated with microbiome variation, it accounted for only a limited portion of the inter-individual differences. Additional factors, such as adverse maternal conditions, prolonged NICU hospitalization, antibiotic exposure, and feeding practices, likely contribute to gut microbiota development [8,63]. Furthermore, environmental influences, including the NICU microbiome and subsequent lifestyle-related exposures, may also shape colonization patterns [64]. These overlapping clinical and environmental factors highlight the multifactorial nature of early life microbial development. Moreover, these factors suggest that GA should be interpreted as a proxy for a complex set of exposures rather than as an isolated determinant. Understanding and managing these influences, particularly in NICU settings, may be crucial for supporting healthy microbiome maturation in vulnerable infants.

The development of the gut microbiota in preterm infants with varying GA was tracked from birth to six months CA, and their progression was compared with that of FT infants. Through this analysis, the dynamic changes in gut microbial composition over time were characterized. Our findings offer new insights into potential microbial interventions tailored to specific GA and developmental stages in preterm infants. For example, the presence of environmental organisms, such as *Ralstonia*, may represent a target for the early modulation of the gut microbiota. A major strength of this study was the longitudinal collection of samples from a large cohort. However, this study had several limitations. First, some samples were excluded from the analysis because of insufficient bacterial DNA or storage issues. Second, the cohort had a relatively low follow-up rate, and the number of fecal samples collected at four- and six-month CA was lower than that of the meconium or samples collected before NICU discharge. This asymmetry in sample size introduces a potential risk of Type II error, particularly in subgroup analyses based on feeding mode, delivery method, or antibiotic exposure. Although cautious conclusions were drawn based on the available data, the results should be interpreted with caution as they may not be generalizable. Third, although potential confounders (feeding type, antibiotics, delivery mode) were not significantly different between the groups, they may still influence the causal inferences or associations drawn in this study. Finally, meconium samples were collected from diapers, which could have led to exposure to environmental microbes. Nevertheless, standardized collection protocols and sterile tools were used to minimize systematic bias.

## 5. Conclusions

Preterm infants have a fragile and vulnerable gut microbial ecosystem, resulting in altered microbiome development compared with that in FT infants. They typically exhibit reduced microbial diversity, delayed colonization by beneficial bacteria, and increased abundance of potential pathogens, which can contribute to adverse health outcomes. Over time, however, gradual maturation of the gut microbiota has been observed, which is marked by increasing alpha diversity and decreasing beta diversity, suggesting greater internal complexity and reduced interindividual variability. Although initial NICU experience exerts a strong influence on early microbiota composition, the gut microbiome of preterm infants tends to converge toward that of FT infants with age. Following NICU discharge, early life exposure continues to shape the microbial community, allowing the gut microbiome to shift from a dysbiotic state toward a more balanced state of symbiosis.

## Figures and Tables

**Table 1:** Maternal and neonatal characteristics of study population.

		VP (<32 wk)	MLP (32≤, <37 wk)	FT (≥37 wk)	*p*	P *		
		[N = 51]	[N = 195]	[N = 93]		VP vs. MLP	VP vs. FT	MLP vs. FT
Maternal characteristics	Assisted pregnancy	13 (25.5%)	66 (33.8%)	11 (11.8%)	<0.001			
	ACS	49 (96.1%)	152 (77.9%)	1 (1.1%)	<0.001			
	PROM > 18 h	13 (25.5%)	30 (15.4%)	4 (4.3%)	0.001			
	HCAM	10 (76.9%)	3 (1.5%)	0 (0.0%)	<0.001			
	maternal DM	13 (25.5%)	28 (14.4%)	7 (7.5%)	0.013			
	oligohydramnios	12 (60.0%)	6 (3.1%)	2 (2.2%)	<0.001			
	Placenta abruption	5 (9.8%)	5 (2.6%)	1 (1.1%)	0.013			
	maternal preeclampsia	20 (39.2%)	50 (25.6%)	3 (3.2%)	<0.001			
	Caesarean section	49 (96.1%)	189 (96.9%)	73 (78.5%)	<0.001			
neonatal characteristics	Gestational age ^a^, week	28.0 ± 2.6	34.1 ± 1.2	37.9 ± 1.0	<0.001	<0.001	<0.001	<0.001
	Birth weight ^a^, g	1125.8 ± 453.5	2186.7 ± 429.7	3160.2 ± 412.5	<0.001	<0.001	<0.001	<0.001
	Male	22 (43.1%)	94 (48.2%)	53 (57.0%)	0.220			
	Requiring intubation at birth	27 (52.9%)	7 (3.9%)	2 (2.2%)	<0.001			
	SGA	14 (27.5%)	18 (9.2%)	7 (7.5%)	0.001			
	RDS	42 (82.4%)	50 (25.6%)	16 (17.2%)	<0.001			
	NEC or FI	12 (23.5%)	20 (10.3%)	3 (3.2%)	0.001			
	BPD	20 (39.2%)	1 (0.5%)	-	<0.001			
	Sepsis	9 (17.6%)	1 (0.5%)	0 (0.0%)	<0.001			
	ROP requiring surgery	11 (21.6%)	0 (0.0%)	-	<0.001			
	TPN duration	46.1 ± 51.3	4.3 ± 3.0	2.2 ± 1.9	<0.001	<0.001	<0.001	1.000
	LOS	101.4 ± 48.0	22.5 ± 13.3	10.5 ± 4.0	<0.001	<0.001	<0.001	<0.001
	AGA at discharge	30 (58.8%)	32 (16.5%)	69 (74.2%)	<0.001			

Values are presented as means with standard deviation for continuous variables ^a^ or frequencies with percentages for categorical variables, as appropriate. *p* value was calculated through χ^2^ test for trend for categorical variables and one-way ANOVA test for continuous variables ^a^. p * value was comparing VP vs. MLP, VP vs. FT, and MLP vs. FT through post-hoc Bonferroni test of one-way ANOVA. Abbreviations: ACS, antenatal corticosteroid; AGA, appropriate for gestational age; BPD, bronchopulmonary dysplasia; DM, diabetes mellitus; FI, feeding intolerance; FT, full term; HCAM, historical chorioamnionitis; LOS, length of stay; MLP, moderate to late preterm; NEC, necrotizing enterocolitis; PROM, premature rupture of membrane; RDS, respiratory distress syndrome; ROP, retinopathy of prematurity; SGA, small for gestational age; TPN, total parenteral nutrition; VP, very preterm.

## Data Availability

The datasets analyzed in this study are not publicly available. The information contained in the data must be protected as confidential and will only become available to those individuals who have obtained permission from the data review board and IRB of Seoul St. Mary’s Hospital to access and use the data for permitted research activities. The original contributions presented in this study are included in the article materials. Further inquiries can be directed to the corresponding authors.

## Supplementary Materials

The following supporting information can be downloaded at: https://www.mdpi.com/article/10.3390/microorganisms13092158/s1. Table S1: The average Relative abundance at Phylum level, Table S2: The average Relative abundance at Genus level; Table S3: Differences in the relative abundance of *Veillonella*; Table S4; *α*- diversities of term and preterm infants at different time points; Table S5: *α-* diversities of term and preterm infants comparison with time.
