# Supplementary material for: Early-Life Development of the Intestinal Microbiome in Preterm and Term Infants Hospitalized in the Neonatal Intensive Care Unit
Source: Microorganisms. 2025 Sep 16;13(9):2158. doi: 10.3390/microorganisms13092158 (PMC12472447; doi:10.3390/microorganisms13092158)
Supplement: Supplementary file 1 [file microorganisms-13-02158-s001.zip › microorganisms-3747951-supplementary.pdf]

Early-Life Development of the Intestinal Microbiome in Preterm and Term Infants Hospitalized in the Neonatal Intensive Care Unit

Jeongmin Shin[1,2], Chang Won Choi[2,3], Hyun-Mi Kang[1], Sae Yun Kim[1*] and Young-Ah Youn[1*]

[1] Department of Pediatrics, Seoul St. Mary's Hospital, College of Medicine, The Catholic University of Korea, 222 Banpo-daero, Seocho-gu, Seoul 06591, Republic of Korea; emmyshin@gmail.com (J.S.), pedhmk@catholic.ac.kr (H.M.K.)

[2] Department of Pediatrics, Seoul National University College of Medicine, Seoul, Republic of Korea; choicw1029@gmail.com (C.W.C.)

[3] Department of Pediatrics, Seoul National University Bundang Hospital, Sungnam, Republic of Korea

* Co-correspondence: sysmile@catholic.ac.kr; Tel: +82 10 3743 3600 (S.Y.K) and lea732@hanmail.net; Tel: +82 10 8760 9597 (Y.-A.Y)

Table S1. The average Relative abundance at Phylum level

	Relative Abundance (%)			*P*	*P**		
Meconium							
	**VP (n=44)**	**MLP (n=127)**	**FT (n=58)**		**VP vs MLP**	**VP vs FT**	**MLP vs FT**
Bacteroidetes	**12.4**	**15.3**	**18.3**	0.052	0.501	0.047	0.374
Firmicutes	**29.9**	**31.9**	**41.2**	0.007	>0.999	0.020	0.014
Proteobacteria	**47.8**	**44.2**	**32.9**	0.032	>0.999	0.054	0.073
Actinobacteria	4.0	5.8	5.9	0.270	0.372	0.503	>0.999
Verrucomicrobia	1.3	0.9	1.1	0.611	0.990	>0.999	>0.999
Others	4.6	1.9	0.7				
Last stool before NICU discharge							
	**VP (n=39)**	**MLP (n=173)**	**FT (n=77)**		**VP vs MLP**	**VP vs FT**	**MLP vs FT**
Bacteroidetes	1.0	1.3	3.3	0.021	>0.999	0.112	0.027
Firmicutes	**29.0**	**49.6**	**53.0**	0.002	0.003	0.002	>0.999
Proteobacteria	**62.3**	**45.1**	**40.1**	0.010	0.030	0.008	0.986
Actinobacteria	**7.6**	**3.8**	**3.4**	0.074	0.100	0.102	>0.999
Verrucomicrobia	0.1	0.1	0.1	0.839	>0.999	>0.999	>0.999
Others	0.1	0.1	0.1				
4 months of corrected age							
	**VP (n=19)**	**MLP (n=66)**	**FT (n=19)**		**VP vs MLP**	**VP vs FT**	**MLP vs FT**
Bacteroidetes	6.0	2.3	0.4	0.150	0.352	0.180	>0.999
Firmicutes	**39.4**	**36.3**	**30.9**	0.415	>0.999	0.587	0.915
Proteobacteria	**27.0**	**34.2**	**30.9**	0.369	0.515	>0.999	>0.999
Actinobacteria	**17.2**	**18.7**	**37.7**	0.005	>0.999	0.021	0.006
Verrucomicrobia	10.3	8.3	0.0	0.174	>0.999	0.277	0.279
Others	0.1	0.3	0.1				
6 months of corrected age							
	**VP (n=17)**	**MLP (n=50)**	**FT (n=20)**		**VP vs MLP**	**VP vs FT**	**MLP vs FT**
Bacteroidetes	4.4	1.0	0.9	0.179	0.232	0.361	>0.999
Firmicutes	**52.7**	**54.6**	**55.4**	0.919	>0.999	>0.999	>0.999
Proteobacteria	**27.4**	**30.9**	**31.3**	0.732	>0.999	>0.999	>0.999
Actinobacteria	**11.3**	**8.8**	**12.2**	0.508	>0.999	>0.999	0.863
Verrucomicrobia	4.0	4.4	0.0	0.367	>0.999	0.937	0.495
Others	0.1	0.3	0.1				

P value was calculated through one-way ANOVA test comparing preterm and full-term infants for continuous variables. *P** value was comparing VP vs MLP, VP vs FT, and MLP vs FT through post-hoc Bonferroni test of one-way ANOVA. Abbreviations: FT, full-term infants; MLP, moderate to late preterm infants; VP, very preterm infants

Table S2. The average Relative abundance at Genus level

	Relative Abundance (%)			*P*	*P**		
Meconium							
	**VP (n=44)**	**MLP (n=127)**	**FT (n=58)**		**VP vs MLP**	**VP vs FT**	**MLP vs FT**
Ralstonia	**16.5**	**13.2**	**8.5**	0.081	0.905	0.087	0.314
Streptococcus	**9.4**	**11.7**	**16.5**	0.006	0.772	**0.007**	0.029
Bacteroides	6.7	**8.5**	**9.8**	0.126	0.522	0.126	0.851
Acidovorax	**8.3**	6.4	5.0	0.274	0.845	0.324	>0.999
Prevotella	3.6	4.4	5.7	0.018	0.717	0.019	0.094
**Bifidobacterium**	3.4	4.4	5.1	0.107	0.484	0.105	0.784
Veillonella	3.1	3.9	4.1	0.671	>0.999	>0.999	>0.999
Staphylococcus	5.8	3.0	3.8	0.314	0.385	>0.999	>0.999
Pelomonas	3.5	3.8	2.4	0.476	>0.999	>0.999	0.678
Sphingomonas	3.3	4.4	1.0	0.204	>0.999	>0.999	0.225
**Faecalibacterium**	1.2	2.4	2.5	0.093	0.139	0.148	>0.999
**Enterococcus**	3.3	1.1	3.3	0.165	0.456	>0.999	0.327
Lachnospiracea_incertae_sedis	1.0	2.2	2.2	0.227	0.407	0.315	>0.999
Escherichia/Shigella	0.9	2.0	2.1	0.645	>0.999	>0.999	>0.999
Sutterella	1.5	1.7	2.2	0.089	>0.999	0.108	0.263
Phenylobacterium	2.4	1.4	1.7	0.365	0.468	>0.999	>0.999
Burkholderia	1.3	1.5	2.0	0.276	>0.999	0.471	0.482
Ureaplasma	4.1	1.2	0.0	0.129	0.333	0.143	>0.999
Akkermansia	1.4	1.0	1.1	0.612	0.991	>0.999	>0.999
Delftia	1.1	0.9	1.0	0.928	>0.999	>0.999	>0.999
Others	18.1	21.1	20.0				
Last stool before NICU discharge							
	**VP (n=39)**	**MLP (n=173)**	**FT (n=77)**		**VP vs MLP**	**VP vs FT**	**MLP vs FT**
Streptococcus	5.8	**18.6**	**22.9**	0.002	0.011	0.002	0.637
**Enterococcus**	6.5	**16.3**	**22.4**	0.006	0.088	0.005	0.237
Enterobacter	**21.2**	**13.4**	7.9	0.018	0.200	0.015	0.285
**Klebsiella**	**10.6**	11.8	**16.6**	0.362	>0.999	0.770	0.577
Escherichia/Shigella	**13.0**	5.0	7.7	0.072	0.074	0.546	0.955
Staphylococcus	1.5	4.8	4.0	0.401	0.534	>0.999	>0.999
Veillonella	6.2	4.2	1.8	0.167	>0.999	0.217	0.495
Clostridium_sensu_stricto	5.4	4.1	0.9	0.058	>0.999	0.130	0.111
**Bifidobacterium**	7.6	2.1	1.7	0.001	0.001	0.002	>0.999
Kluyvera	4.8	2.6	0.2	0.008	0.331	0.009	0.080
Raoultella	3.0	1.7	0.5	0.049	0.484	0.050	0.308
Bacteroides	0.6	0.7	2.6	0.010	>0.999	0.088	0.011
Citrobacter	0.3	1.4	1.0	0.781	>0.999	>0.999	>0.999
Acinetobacter	0.0	1.6	0.3	0.351	0.838	>0.999	0.702
Others	13.6	11.8	9.5				
4 months of corrected age							
	**VP (n=19)**	**MLP (n=66)**	**FT (n=19)**		**VP vs MLP**	**VP vs FT**	**MLP vs FT**
**Bifidobacterium**	**16.9**	**18.7**	**35.6**	0.011	>0.999	0.033	0.013
Veillonella	**19.4**	**24.3**	**12.5**	0.054	0.963	0.786	0.054
Escherichia/Shigella	10.3	**13.5**	**12.6**	0.765	>0.999	>0.999	>0.999
Akkermansia	**10.5**	8.6	0.0	0.176	>0.999	0.290	0.272
**Klebsiella**	4.5	4.5	3.4	0.852	>0.999	>0.999	>0.999
Enterobacter	3.3	3.5	6.0	0.422	>0.999	0.858	0.621
Citrobacter	0.5	3.9	2.2	0.098	0.118	>0.999	0.865
**Enterococcus**	1.9	2.1	3.8	0.227	>0.999	0.446	0.309
Clostridium_sensu_stricto	2.3	2.7	1.1	0.252	>0.999	0.949	0.293

Bacteroides	3.8	1.7	0.3	0.366	0.879	0.488	>0.999
Clostridium_XlVa	1.8	1.3	2.4	0.602	>0.999	>0.999	0.978
Lactobacillus	3.0	0.7	0.6	0.169	0.208	0.387	>0.999
Ralstonia	0.2	1.3	0.7	0.646	>0.999	>0.999	>0.999
Flavonifractor	4.0	0.3	0.4	<0.001	<0.001	<0.001	<0.001
Others	17.6	13.1	18.4				
6 months of corrected age							
	**VP (n=17)**	**MLP (n=50)**	**FT (n=20)**		**VP vs MLP**	**VP vs FT**	**MLP vs FT**
Veillonella	**24.6**	**37.1**	**35.8**	0.062	0.060	0.230	>0.999
Escherichia/Shigella	**10.3**	**17.0**	7.3	0.066	0.471	>0.999	0.091
**Bifidobacterium**	**10.9**	**8.8**	**12.3**	0.510	>0.999	>0.999	0.811
Clostridium_sensu_stricto	5.3	5.2	5.9	0.941	>0.999	>0.999	>0.999
Haemophilus	1.6	3.6	**7.8**	0.061	>0.999	0.074	0.172
Akkermansia	4.0	4.6	0.0	0.365	>0.999	0.956	0.489
**Klebsiella**	7.7	2.4	2.2	0.024	0.080	0.063	>0.999
Lachnospiracea_incertae_sedis	2.8	2.3	2.1	0.932	>0.999	>0.999	>0.999
Enterobacter	1.4	1.5	5.0	0.036	>0.999	0.124	0.041
Clostridium_XlVa	3.5	1.7	1.8	0.391	0.549	0.834	>0.999
Flavonifractor	5.6	1.5	0.5	0.000	0.001	<0.001	>0.999
Citrobacter	1.1	2.1	1.5	0.527	0.869	>0.999	>0.999
**Enterococcus**	3.1	1.4	0.7	0.335	0.694	0.460	>0.999
Bacteroides	4.3	0.4	0.2	0.035	0.042	0.083	>0.999
Others	13.5	10.6	16.9				

P value was calculated through one-way ANOVA test comparing preterm and full-term infants for continuous variables[a]. *P** value was comparing VP vs MLP, VP vs FT, and MLP vs FT through post-hoc Bonferroni test of one-way ANOVA.
Abbreviations: FT, full-term infants; MLP, moderate to late preterm infants; VP, very preterm infants

Table S3. Differences in the relative abundance of *Veillonella*

	Mec	N_{dc}	4m	6m	P	*Post-hoc camparison*						P value by Friedman test
						P^a	P^b	P^c	P^d	P^e	P^f	
						Mec vs. N_{dc}	*Mec vs. 4m*	*Mec vs. 6m*	N_{dc} *vs. 4m*	N_{dc} *vs. 6m*	*4m vs. 6m*	
VP	3.1	6.2	19.4	24.6	<0.001	>0.999	<0.001	<0.001	0.002	<0.001	>0.999	0.011
MLP	3.9	4.2	24.3	37.1	<0.001	>0.999	<0.001	<0.001	<0.001	<0.001	<0.001	<0.001
FT	4.1	1.8	12.5	35.8	<0.001	>0.999	0.031	<0.001	0.002	<0.001	<0.001	<0.001

The gut microbiome was analyzed at four time points: meconium (Mec), the last stool sample before NICU discharge (N_{dc}), and stool samples collected at 4 months (4m) or 6 months (6m) of corrected age (for preterm infants) or chronological age (for term infants). *P* value were calculated by Friedman test.

Table S4. α**- diversities of term and preterm infants at different time points**

Meconium						
VP (n=44)	MLP (n=127)	FT (n=58)	*P*	VP vs MLP	VP vs FT	MLP vs FT
2.523 (1.976-3.339)	2.633 (2.104-3.444)	2.895 (2.445-3.487)	0.068			
Last stool before nicu discharge						
VP (n=39)	MLP (n=173)	FT (n=77)	*P*	VP vs MLP	VP vs FT	MLP vs FT
1.345 (0.938-1.601)	1.308 (0.954-1.618)	1.064 (0.823-1.391)	0.009	>0.999	0.078	0.010
4 months of corrected age						
VP (n=19)	MLP (n=66)	FT (n=19)	*P*	VP vs MLP	VP vs FT	MLP vs FT
1.849 (1.574-2.028)	1.68 (1.328-1.946)	1.603 (1.311-1.893)	0.224			
6 months of corrected age						
VP (n=17)	MLP (n=50)	FT (n=20)	*P*	VP vs MLP	VP vs FT	MLP vs FT
1.995 (1.844-2.344)	1.667 (1.5-1.95)	1.949 (1.709-2.143)	0.001	0.004	>0.999	0.010

Values are presented as median (IQR). *P* value was calculated through Independent-Samples Kruskal-Wallis Test

Abbreviations: FT, full-term infants; IQR, interquartile range; MLP, moderate to late preterm infants; VP, very preterm infant

Table S5. α- diversities of term and preterm infants comparison with time

**Mec**	**N_{dc}**	**4m**	**6m**	***P***	***Post-hoc analyses***					
					P^a	***P^b***	***P^c***	***P^d***	***P^e***	***P^f***
**VP**										
**[N=44]**	**[N=39]**	**[N=19]**	**[N=17]**	***P***	***P^a***	***P^b***	***P^c***	***P^d***	***P^e***	***P^f***
2.523 (1.976-3.339)	1.345 (0.938-1.601)	1.849 (1.574-2.028)	1.995 (1.844-2.344)	<0.001	<0.001	0.078	>0.999	0.039	0.001	>0.999
**MLP**										
**[N=127]**	**[N=173]**	**[N=66]**	**[N=50]**	***P***	***P^a***	***P^b***	***P^c***	***P^d***	***P^e***	***P^f***
2.633 (2.104-3.444)	1.308 (0.954-1.618)	1.68 (1.328-1.946)	336 (292-384)	<0.001	<0.001	<0.001	<0.001	<0.001	0.001	>0.999
**FT**										
**[N=58]**	**[N=77]**	**[N=19]**	**[N=20]**	***P***	***P^a***	***P^b***	***P^c***	***P^d***	***P^e***	***P^f***
2.895 (2.445-3.487)	1.064 (0.823-1.391)	1.603 (1.311-1.893)	1.949 (1.709-2.143)	<0.001	<0.001	<0.001	0.068	0.042	<0.001	>0.999

Values are presented as median (IQR). *P* values were calculated with the Kruskal-Wallis test across four sampling time points (meconium [Mec], last stool before NICU discharge [N_{dc}], 4 months corrected age [4m], and 6 months corrected age [6m]). The post-hoc analyses were conducted: P^a (Mec vs. N_{dc}), P^b (Mec vs. 4m), P^c (Mec vs. 6m), P^d (N_{dc} vs. 4m), P^e (N_{dc} vs. 6m), and P^f (4m vs. 6m). Abbreviations: FT, full-term infants; IQR, interquartile range; MLP, moderate to late preterm infants; NICU, neonatal intensive care unit; VP, very preterm infant
